# Superiority and non-inferiority: two sides of the same coin?

**DOI:** 10.1186/s13063-018-2885-z

**Published:** 2018-09-17

**Authors:** David T. Dunn, Andrew J. Copas, Peter Brocklehurst

**Affiliations:** 1 MRC Clinical Trials Unit at UCL, 90 High Holborn, London, WC1V 6LJ UK; 2 London Hub for Trials Methodology Research, MRC Clinical Trials Unit at UCL, London, UK; 3 Birmingham Clinical Trials Unit, University of Birmingham, Birmingham, UK

**Keywords:** Guidelines, Regulatory, Delta, Superiority, Non-inferiority

## Abstract

**Background:**

The classification of phase 3 trials as superiority or non-inferiority has become routine, and it is widely accepted that there are important differences between the two types of trial in their design, analysis and interpretation.

**Main text:**

There is a clear rationale for the superiority/non-inferiority framework in the context of regulatory trials. The focus of our article is non-regulatory trials with a public health objective. First, using two examples from infectious disease research, we show that the classification of superiority or non-inferiority trials is not always straightforward. Second, we show that several arguments for different approaches to the design, analysis and interpretation of superiority and non-inferiority trials are unconvincing when examined in detail. We consider, in particular, the calculation of sample size (and the choice of delta or the non-inferiority margin), intention-to-treat versus per-protocol analyses, and one-sided versus two-sided confidence intervals. We argue that the superiority/non-inferiority framework is not just unnecessary but can have a detrimental effect, being a barrier to clear scientific thought and communication. In particular, it places undue emphasis on tests for significance or non-inferiority at the expense of estimation. We emphasise that these concerns apply to phase 3 non-regulatory trials in general, not just to those where the classification of the trial as superiority or non-inferiority is ambiguous.

**Conclusions:**

Guidelines and statistical practice should abandon the sharp division between superiority and non-inferiority phase 3 non-regulatory trials and be more closely aligned to the clinical and public health questions that motivate the trial.

## Background

It is widely accepted that important differences exist between superiority and non-inferiority trials in terms of their design, analysis and interpretation. This is reflected in regulatory agency guidelines, CONSORT statements on the reporting of trials and review articles [1–5]. The European Medicines Agency states the “*pre-definition of a trial as a superiority trial, an equivalence trial or a non-inferiority trial is necessary for numerous reasons*” [4], and one reporting guideline asserts that non-inferiority trials present *“particular difficulties in their design, analysis, and interpretation*” [2]. Focussing on non-regulatory trials with a public health objective, our article challenges this dogma.

## Non-inferiority or superiority, which is it?

CAP-IT is a UK-based factorial randomised controlled trial assessing the optimal dose and duration of amoxicillin treatment for children with community-acquired pneumonia, with a primary outcome of clinical non-response requiring re-treatment (http://www.nets.nihr.ac.uk/projects/hta/138811). In original discussions, it was decided to compare the doses of 125 mg and 250 mg, both three times per day (although the final trial design was based on weight-band dependent dosing). At that time, and in the absence of any randomised evidence, the British National Formulary specified a 250 mg dose, but surveys had shown that the 125 mg dose was more commonly used in clinical practice [6, 7]. This raised the dilemma of which dose should be defined as standard and which as experimental. Following conventional statistical thinking, defining 250 mg as the standard dose implies a non-inferiority trial as the lower 125 mg dose would be unlikely to reduce the rate of relapse; conversely, defining 125 mg as the standard dose implies a superiority trial. The fact that the definition of standard versus experimental dose is arbitrary implies that the classification of the trial as superiority or non-inferiority is also arbitrary.

Even when one treatment clearly represents the standard intervention, the definition of the trial as non-inferiority or superiority may be a moot point. In the public health approach to HIV treatment, the recommended second-line regimen is a boosted protease inhibitor in combination with two nucleoside reverse transcriptase inhibitors (NRTIs) [8]. NRTIs are also used in first-line regimens and concern exists that viral cross-resistance will render them only partially effective. Two similar trials of second-line therapy were therefore conducted (EARNEST, SECOND-LINE) [9, 10], in which participants were randomised to receive either clinician-selected NRTIs or raltegravir (an integrase inhibitor). EARNEST was conducted in sites in sub-Saharan Africa, whereas the sites in SECOND-LINE were more diverse, with low-, middle- and high-income countries represented (Table 1). Although both studies regarded the NRTI group as standard and the raltegravir group as experimental, SECOND-LINE was defined as a non-inferiority trial and EARNEST as a superiority trial (Table 1). As raltegravir was more expensive at the time of the trial than NRTIs, the EARNEST investigators argued it would need to be shown to be more effective than NRTIs. The SECOND-LINE investigators adopted a more modest aim of demonstrating that raltegravir, which has a favourable toxicity profile, was an acceptable alternative to NRTIs that widened the range of therapeutic options. The results and interpretation of these two studies are discussed later.

**Table 1:** Comparison of SECOND-LINE and EARNEST studies

Study	SECOND-LINE [9]	EARNEST [10]
Design	Non-inferiority	Superiority^a^
Investigators’ rationale	Raltegravir less toxic than nucleoside reverse transcriptase inhibitors (NRTIs), aim to show similar efficacy	Raltegravir more expensive, aim to show better efficacy than NRTIs
Setting	37 sites in 15 countries in 5 continents	14 sites in 5 sub-Saharan African countries
Number of subjects	588	859
Delta/non-inferiority margin	12%	10%
Primary endpoint	Viral load < 200 copies/mL at 48 weeks	Composite endpoint (good HIV disease control) at 96 weeks
Frequency of primary endpoint	81% NRTI 83% raltegravir Difference = 1.8% (95% CI –4.7 to 8.3)	60% NRTI 64% raltegravir Difference = 4.2% (95% CI –2.4 to 10.7)
Conclusion	Criterion for non-inferiority fulfilled	Superiority of raltegravir not shown
Interpretation (précised from paper Abstract)	The raltegravir regimen was easy to administer, effective, safe and tolerable … This simple NRTI-free treatment strategy might extend the successful public health approach to management of HIV	NRTIs retained substantial virologic activity without evidence of increased toxicity, and there was no advantage to replacing them with raltegravir

^a^The EARNEST trial had a third arm – protease inhibitor monotherapy – but this is not relevant to the comparison with SECOND-LINE and is not presented here

In the remaining sections we discuss some areas where important differences are perceived to exist between trials classified as non-inferiority and those classified as superiority. Our points apply both to trials where the classification is natural and those where it is not, such as the CAP-IT trial.

## Sample size and choice of non-inferiority margin

In a superiority trial, the sample size calculation is conventionally based on achieving adequate power to demonstrate that the relevant confidence limit for the difference between the two treatments excludes zero, assuming that the experimental treatment is superior by a given amount (‘delta’). In a non-inferiority trial, the calculation is conventionally based on achieving adequate power to demonstrate that the relevant confidence limit excludes the specified non-inferiority margin, assuming that the two treatments are equally effective [5, 11]; these problems are symmetrical, given these assumptions [11]. In the case of continuous variables, the sample size formulae are identical, provided two-sided confidence intervals (CIs) are used. In the case of binary variables, the formulae yield minor differences related to the computation of standard errors; this difference can go in either direction [12].

This raises the critical question of whether delta and the non-inferiority margin are conceptually different or identical. We believe they are the same, with their meaning best captured by the term ‘smallest clinically important difference’, which can be quantified by eliciting opinions of expert clinicians and patients [13, 14]. There is no good reason why the size of this difference (and by implication the sample size) should depend on whether the trial is defined as superiority or non-inferiority. In particular, it is a misconception that non-inferiority trials need to be much larger than superiority trials [12]. One reason why superiority trials are sometimes smaller is that delta is instead chosen as the value that corresponds to the *expected* difference, with optimistic values selected to reduce the sample size [14, 15]. Additionally, some non-inferiority trials define the non-inferiority margin as a certain fraction of the effect of the standard treatment (active control) as estimated from previous placebo-controlled trials [1, 16]. However, the logic of this approach has been challenged in the regulatory setting [17]. The rationale for triangulating results with a hypothetical placebo group is even weaker in a health service context if offering no treatment to a patient with the condition in question is not a viable clinical option.

## Intention-to-treat versus per-protocol analyses

In superiority trials, a rigorous primary analysis should include all randomised patients, irrespective of whether they took study medication as randomised (intention-to-treat). Historically, non-inferiority trials placed greater emphasis on ‘per-protocol’ analyses, which exclude patients with major protocol violations, including unacceptably low levels of adherence to the study drug [18]. The rationale for this is that including such patients dilutes the observed difference between the randomised groups and therefore increases the chance of demonstrating non-inferiority (if the experimental treatment is inferior). However, there is increasing scepticism about the value of per-protocol analyses because these subvert the integrity of the randomisation and the considerable variation in interpretation of what constitutes the per-protocol population [15, 19–21]. A range of methods to assess the impact of non-adherence have been developed, which can be applied equally to superiority and non-inferiority trials [22, 23]. The selection of the most appropriate method depends critically on the primary research question (e.g. whether inference is intended to apply to all patients or just to those who adhere to the recommended treatment), requiring clear communication between clinical researchers and statisticians [22].

## Significance tests versus confidence intervals (CIs)

In the SECOND-LINE trial described above, the non-inferiority margin was specified as 12%. Further, 80.8% of patients in the NRTI (control) group and 82.6% of patients in the raltegravir (experimental) group met the primary endpoint (HIV RNA plasma viral load < 200 copies/mL at 48 weeks), a difference of 1.8% (95% CI –4.7 to 8.3). In the Abstract, the authors concluded that the “*criterion for non-inferiority was fulfilled*” [2] i.e. following advice in the CONSORT guidelines to take the non-inferiority hypothesis (margin) into account in the interpretation of the results. However, the lower limit of the observed CI tells us that raltegravir is inferior to NRTIs by a margin of 4.7% at most, i.e. approximately three-fold smaller than the pre-specified non-inferiority margin. As inference should be based primarily on point estimates and CIs rather than significance tests [24], the emphasis in the results should be on the observed value of 4.7% rather than the arbitrary value of 12%. As other authors have pointed out: “*we will eventually come to see that the pre-specification by the sponsor of a non-inferiority margin does not form part of any rational approach to analysing such trials*” [25]. Finally, reports of superiority trials usually mention ‘delta’ only in the justification of the sample size calculation in the Methods section, rarely playing a part in the interpretation of the results. This is in sharp contrast with the central role of the non-inferiority margin in the interpretation of non-inferiority trials, and is a logical inconsistency between the two types of trial.

## One-sided or two-sided confidence intervals (CIs)

A leading medical journal requires that superiority trials present two-sided CIs but that non-inferiority trials present one-sided CIs [26]. This is based on the dubious argument that *“a non-inferiority trial only aims to demonstrate non-inferiority and does not aim to distinguish non-inferiority from superiority*” [26]. However, regulatory agencies do not exclude the possibility of switching between superiority and non-inferiority [4], and it makes no sense to ignore evidence on superiority if a trial produces such evidence, even if this outcome was not anticipated. A recent paper argues that a clear distinction should be made between statistical and clinical superiority, along with consistent presentation of two-sided CIs [11].

## Same results, different conclusions

The SECOND-LINE and EARNEST trials both found no material difference between the two randomised treatment strategies in terms of the study primary endpoints (Table 1). The investigators of EARNEST (the superiority trial) interpreted their results as evidence supporting the use of NRTIs in second-line regimens; the investigators of SECOND-LINE (the non-inferiority trial) concluded that raltegravir was an acceptable alternative to NRTIs in a second-line regimen. These conclusions are both ‘correct’ within the particular statistical framework chosen by the trial investigators. The fact that the conclusions are contradictory, despite a partial geographical overlap in the location of trial sites, raises concerns about the framework itself. While it is not unreasonable for two scientists to interpret the same data differently, the pre-definition of a trial as superiority or non-inferiority tends to impel a certain narrative influenced by the results of tests of significance or non-inferiority.

## Decision-making

Non-inferiority trials were originally developed in the setting of drug approval, where regulatory agencies have to make a binary decision – either to licence or to not licence the experimental treatment. To ensure that the process is transparent and explicit, the agencies justifiably require that the study sponsors produce detailed study protocols, including pre-specification of the non-inferiority margin. In contrast, the main objective of non-licencing trials is to publish information that allows other bodies (commissioners of health services, producers of clinical guidelines, etc.) to make considered decisions about which treatments should be funded or recommended. These decisions are complex and need to consider issues such as cost, adverse drug effects and quality of life, in addition to clinical efficacy [12]. Ideally, decision analysis models should be employed based on a synthesis of all relevant evidence. Evidence syntheses do not treat superiority and non-inferiority trials differently, nor do they consider whether a trial delivered a significant or non-significant result. As pointed out by Claxton: “*the historical accident that dictates which of the alternatives is regarded as current practice is irrelevant*” [27].

## Conclusions

Our two examples highlight that the classification of trials as superiority or non-inferiority is sometimes arbitrary, particularly when the classification of treatment groups as standard or experimental is not straightforward. This would not matter much if the distinction was only one of terminology, but the received wisdom is that this classification has an important bearing on how a trial is designed, analysed and interpreted. However, we have shown that the arguments in support of this belief are weak and contend that the superiority/non-inferiority framework can act as a barrier to clear scientific thought and communication. In particular, it places undue emphasis on tests for significance or non-inferiority at the expense of estimation. We stress that these concerns apply to phase 3 non-regulatory trials in general, not just to those where the classification is ambiguous. Guidelines and statistical practice should abandon the sharp division between superiority and non-inferiority phase 3 non-regulatory trials, and should instead be more closely aligned to the clinical and public health questions that motivate the trial.

## References

[1] U.S. Department of Health and Human Services, Food and Drug Administration. Non-inferiority clinical trials to establish effectiveness. In: Guidance for industry; 2016. https://www.fda.gov/downloads/Drugs/Guidances/UCM202140.pdf. Accessed 11 Sept 2018.

[2] Piaggio G, Elbourne DR, Altman DG, Pocock SJ, Evans SJ (2006). Reporting of noninferiority and equivalence randomized trials: an extension of the CONSORT statement. JAMA.

[3] ICH Harmonised Tripartite Guideline. Choice of control group and related issues in clinical trials. E10. 2000. https://www.ich.org/fileadmin/Public_Web_Site/ICH_Products/Guidelines/Efficacy/E10/Step4/E10_Guideline.pdf. Accessed 11 Sept 2018.

[4] The European Agency for the Evaluation of Medicinal Products, Committee for Proprietary Medicinal Products. Points to consider on switching between superiority and non-inferiority. 2000. http://www.ema.europa.eu/docs/en_GB/document_library/Scientific_guideline/2009/09/WC500003658.pdf. Accessed 11 Sept 2018.

[5] Mauri L, D'Agostino RB (2017). Challenges in the design and interpretation of noninferiority trials. N Engl J Med.

[6] Bielicki JA, Barker CI, Saxena S, Wong IC, Long PF, Sharland M (2015). Not too little, not too much: problems of selecting oral antibiotic dose for children. BMJ.

[7] Saxena S, Ismael Z, Murray ML, Barker C, Wong IC, Sharland M, Long PF (2014). Oral penicillin prescribing for children in the UK: a comparison with BNF for children age-band recommendations. Br J Gen Pract.

[8] World Health Organization (2013). Consolidated guidelines on the use of antiretroviral drugs for treating and preventing HIV infection: recommendations for a public health approach.

[9] Second-line Study Group (2013). Ritonavir-boosted lopinavir plus nucleoside or nucleotide reverse transcriptase inhibitors versus ritonavir-boosted lopinavir plus raltegravir for treatment of HIV-1 infection in adults with virological failure of a standard first-line ART regimen (SECOND-LINE): a randomised, open-label, non-inferiority study. Lancet.

[10] Paton NI, Kityo C, Hoppe A, Reid A, Kambugu A, Lugemwa A, van Oosterhout JJ, Kiconco M, Siika A, Mwebaze R (2014). Assessment of second-line antiretroviral regimens for HIV therapy in Africa. N Engl J Med.

[11] Ganju J, Rom D (2017). Non-inferiority versus superiority drug claims: the (not so) subtle distinction. Trials.

[12] Blackwelder WC (1982). “Proving the null hypothesis” in clinical trials. Control Clin Trials.

[13] Corica T, Joseph D, Saunders C, Bulsara M, Nowak AK (2014). Intraoperative radiotherapy for early breast cancer: do health professionals choose convenience or risk?. Radiat Oncol.

[14] Spiegelhalter DJ, Freedman LS, Parmar MKB (1994). Bayesian approaches to randomized trials. J R Stat Soc Ser A.

[15] Fleming TR (2008). Current issues in non-inferiority trials. Stat Med.

[16] Schumi J, Wittes JT (2011). Through the looking glass: understanding non-inferiority. Trials.

[17] Snapinn S, Jiang Q (2008). Preservation of effect and the regulatory approval of new treatments on the basis of non-inferiority trials. Stat Med.

[18] Jones B, Jarvis P, Lewis JA, Ebbutt AF (1996). Trials to assess equivalence: the importance of rigorous methods. BMJ.

[19] Hill A, Sabin C (2008). Designing and interpreting HIV noninferiority trials in naive and experienced patients. AIDS.

[20] Abraha I, Montedori A (2010). Modified intention to treat reporting in randomised controlled trials: systematic review. BMJ.

[21] Wiens BL, Zhao W (2007). The role of intention to treat in analysis of noninferiority studies. Clin Trials.

[22] Shrier I, Steele RJ, Verhagen E, Herbert R, Riddell CA, Kaufman JS (2014). Beyond intention to treat: what is the right question?. Clin Trials.

[23] Hauck WW, Anderson S (1999). Some issues in the design and analysis of equivalence trials. Drug Inf J.

[24] Sterne JA, Davey Smith G (2001). Sifting the evidence-what's wrong with significance tests?. BMJ.

[25] Senn S (2005). Equivalence is different - some comments on therapeutic equivalence. Biom J.

[26] Kaji AH, Lewis RJ (2015). Noninferiority trials: is a new treatment almost as effective as another?. JAMA.

[27] Claxton K (1999). The irrelevance of inference: a decision-making approach to the stochastic evaluation of health care technologies. J Health Econ.

